# Supplementary material for: The Strength of Structural Diversity in Online Social Networks
Source: Research (Wash D C). 2021 May 26;2021:9831621. doi: 10.34133/2021/9831621 (PMC8328400; doi:10.34133/2021/9831621)
Supplement: Supplementary Materials — Figure S1: data collection procedure of an ego network. Figure S2: gender distribution. Figure S3: illustration of the function of social bridges in an example ego network. Figure S4: examples of social bridges in ego networks. Table S1: data descriptions of three popularity measures. Table S2: correlation coefficients between three popularity measures. Table S3: data descriptions of other activity-related factors. Table S4: data descriptions of indegree, weak diversity measure, and strong diversity measure. Table S5: correlation coefficients between indegree, weak diversity measure, and strong diversity measure. Table S6: covariates accounted for in propensity score matching. [file 9831621.f1.pdf]

SUPPLEMENTARY MATERIALS:
The Strength of Structural Diversity in Online Social Networks

Yafei Zhang[1,2], Lin Wang[1], Jonathan J. H. Zhu[2,*], Xiaofan Wang[1,3,*], Alex 'Sandy' Pentland[4]

1. Shanghai Jiao Tong University; 2. City University of Hong Kong;
3. Shanghai University; 3. Massachusetts Institute of Technology

Note 1 - Data collection

Online social platforms create quasi-real social systems, where users can express their opinions, share experiences, and maintain relationships through various online behaviors such as posting, commenting, and voting. In recent years, the emergence and rapid proliferation of social platforms provide unprecedented opportunities to investigate a range of social and economic problems at a large scale which are generally not possible with traditional one-time, self-reported data.

Using data from Zhihu[1], a Chinese knowledge-sharing website which operates in a way similar to Quora[2], we present an exploratory investigation of how the structural diversity among one's social neighbors could be utilized to predict his/her online social reputation. The network data can be classified into three main types: social network data, social reputation data, and activity data. Starting from a randomly selected user, the social network data are collected in a snowball sampling manner. Figure S1 illustrates the collection procedure of an ego network (only users that are one hop away from the ego user are shown). For the ego user (denoted by A) located in the hub of a wheel, we collect his/her complete follower and followee lists, and the followee lists of his/her followers. The ego network is then constructed based on the explicit social ties between all users covered in the procedure. In total, we collect 234,834 ego users, but to construct these 234,834 ego networks, millions of users are covered. Social reputation data include how many upvotes, thanks, and favorites the ego users have received on the platform. We further collect informative activity data of these 234,834 ego users, including how many questions they asked and answered, followed topics and questions, along with some other kinds of publicly available data. All the collected data are based on public information on the platform and don't include any users with privacy restrictions.

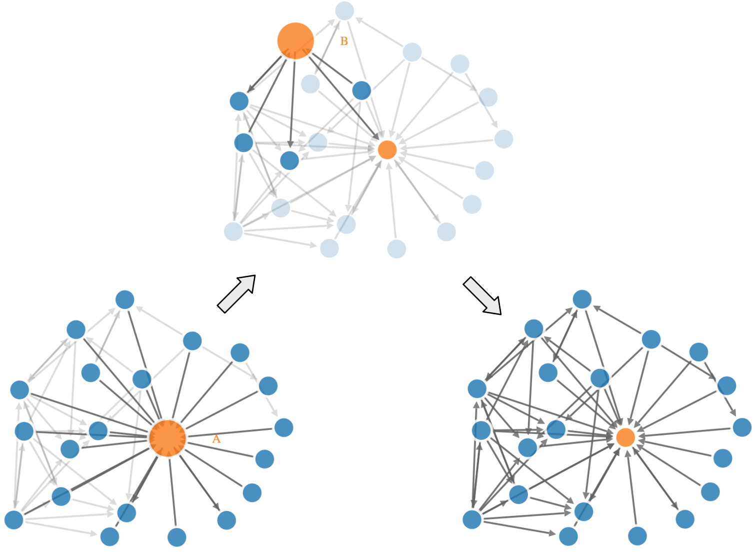

Figure S1. Data collection procedure of an ego network.

[1] https://www.zhihu.com
[2] https://www.quora.com

Note 2 - Data description

1) Social reputation data

On the knowledge-sharing website, how many upvotes, thanks, and favorites one has received can be viewed as indicators of his/her popularity on the platform. There are 234,834 ego users in total, but nearly two-thirds of them receive no upvotes, three-quarters receive no thanks and three-quarters receive no favorites.

Table S1 shows the basic descriptions of the three popularity measures, where the number of upvotes that an ego user has received could be as many as 1,946,788, the number of thanks that an ego user has received could be as many as 234,912, and the number of favorites that an ego user has received could be as many as 2,376,039. Table S2 presents the pairwise Pearson correlation coefficients between the three popularity measures. As described in the Methods section in the main text, using nonnegative matrix factorization (NMF), these three popularity measures are then collapsed into a single measure (termed *Social Reputation Index*) to comprehensively depict one's social reputation online.

2) Activity data

Table S3 shows the basic descriptions of some other activity-related data. The upper panel displays self-generated data which may indicate one's activity level on the platform. Answer count means how many answers an individual has contributed to the platform; question count means how many questions one has asked on the platform; article count means how many articles one has written on the platform; column count means how many special columns one has written on the platform; pin count means how many personal posts one has posted on the platform; favorite count means how many favorite lists one has created for her/him-self on the platform. The lower panel displays how many questions, topics, columns, and favorite lists (generally generated by other users) that an individual has followed on the platform, which may indicate one's personal interests on the platform. Specifically, followed column count means how many columns one has followed on the platform; followed favorite count means how many favorite lists one has followed on the platform; followed question count means how many questions one has followed on the platform; followed topic count means how many topics one has followed on the platform. As shown in the table, the data distributions are more or less skewed.

3) Gender

On the platform, users may choose to disclose their gender or not. Figure S2 shows the distribution of the self-reported gender, where "NA" means the group of ego users whose gender is not known. Specifically, 87,213 of 234,834 (37%) ego users don't disclose their gender at all, 80,071 of 234,834 (34%) users are female users and 67,550 of 234,834 (29%) users are male users.

Table S1. Data descriptions of three popularity measures. The corresponding Chinese meaning of each variable is shown in square brackets.

	count	mean	std	min	25%	50%	75%	max
Upvotes [赞同]	234834	262.815	8678.739	0	0	0	2	1946788
Thanks [感谢]	234834	39.766	1187.614	0	0	0	0	234912
Favorites [收藏]	234834	126.171	6383.124	0	0	0	0	2376039

Table S2. Correlation coefficients between three popularity measures. The p values for all the pairwise Pearson correlations in the table are p<0.001, N=234,834 obs.

	Upvotes	Thanks	Favorites
Upvotes	1.000		
Thanks	0.921	1.000	
Favorites	0.489	0.545	1.000

Table S3. Data descriptions of other activity-related factors. The corresponding Chinese meaning of each variable is shown in square brackets.

	count	mean	std	min	25%	50%	75%	max
Answer count [回答数]	234834	7.299	51.312	0	0	0	3	8533
Question count [提问数]	234834	0.783	4.307	0	0	0	1	934
Article count [文章数]	234834	0.346	8.980	0	0	0	0	1539
Column count [专栏数]	234834	0.012	0.134	0	0	0	0	14
Pin count [想法数]	234834	0.537	11.551	0	0	0	0	2184
Favorite count [收藏数]	234834	3.893	6.597	0	0	1	5	84
Followed column count [关注的专栏数]	234834	4.970	17.597	0	0	1	4	1274
Followed favorite count [关注的收藏夹数]	234834	3.143	15.799	0	0	0	1	1689
Followed question count [关注的问题数]	234834	80.013	426.580	0	1	9	49	101094
Followed topic count [关注的话题数]	234834	30.995	68.458	0	6	16	33	9080

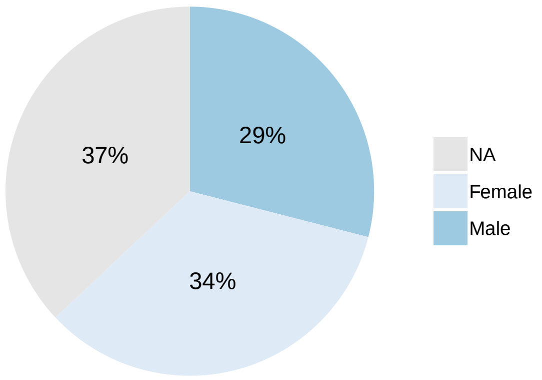

Figure S2. Gender distribution.

Note 3 - Weak and strong connectivity

To depict one's structural diversity, we first propose two diversity measures, which we term weak diversity measure and strong diversity measure, based on weak and strong connectivity patterns among one's connected neighbors. As described in the main text, indegree is directly measured by the number of followers of an ego user whereas weak diversity measure and strong diversity measure are computed by the number of weakly and strongly connected components in the connected neighborhood (formed by followers) of the ego user, respectively.

Table S4 presents the basic descriptive analysis of indegree, weak diversity measure, and strong diversity measure. Nearly half of the ego users in the sample have no followers at all, about 14% ego users have only one follower and the majority (about 87%) of ego users have less than 10 followers. However, some users could have as many as tens of thousands of followers. For example, one user has 654,497 followers, which also indicates the high inequality of the number of followers on the platform. Similar patterns are also found in terms of weak and strong diversity measures. Table S5 further presents the pairwise Pearson correlation coefficients between indegree, weak diversity measure, and strong diversity measure. Inevitably, both of them are somewhat highly correlated since weak diversity measure and strong diversity measure are built upon the number of followers.

Table S4. Data descriptions of indegree, weak diversity measure, and strong diversity measure.

	count	mean	std	min	25%	50%	75%	max
Indegree	234834	83.136	3634.921	0	0	1	3	654497
Weak diversity measure	234834	26.437	642.707	0	0	1	3	156839
Strong diversity measure	234834	68.658	2765.348	0	0	1	3	459616

Table S5. Correlation coefficients between indegree, weak diversity measure, and strong diversity measure. The p values for all the pairwise Pearson correlations in the table are p<0.001, N=234,834 obs.

	Indegree	Weak diversity measure	Strong diversity measure
Indegree	1.000		
Weak diversity measure	0.763	1.000	
Strong diversity measure	0.992	0.815	1.000

Note 4 - Social bridges

From the view of the link-prediction problems in network science, individuals who have many common neighbors are also more likely to establish social connections between them. For example, in the collaboration network, the probability that two scientists will collaborate in the future is likely to increase with the number of collaborators they have in common. In directed networks, we take shared followees of two individuals into consideration since two individuals who share lots of followees would tend to be similar to each other. We term the shared followees *social bridges* as they could function as implicit social ties "linking" unconnected individuals or social components.

Figure S3 illustrates how social bridges work in a given ego network. As shown in the figure, user 1 and user 2 share a large proportion of followees, therefore they may belong to one social component even without a direct social tie between them. Figure S4 gives four more examples. Panel (a) and panel (b) illustrate two examples where no direct social connections exist between any two followers (colored in blue) of respective ego users (colored in orange), while panel (c) and panel (d) illustrate another two examples where few social connections exist between the followers (colored in blue) of respective ego users (colored in orange). In these cases, diversity measures which only consider direct social connections between followers, such as weak or strong diversity measure, may fail to take the potential similarity of users into consideration, but social bridges provide additional power to depict the implicit structural diversity

As we have described in the main text, for any two followers of a given ego user, whether there exists a "bridged connection" between them is determined by the Jaccard similarity of their followee sets: $JaccardSim(i,j) = |F_i \cap F_j|/|F_i \cup F_j|$. For example, in the example network of Figure S3, the Jaccard similarity of user 1 and user 2's followees is $JaccardSim(user1, user2) = 0.48$, while the Jaccard similarity of user 3 and user 4's followees is $JaccardSim(user3, user4) = 0.12$. When the threshold of Jaccard similarity is set as 0.2, there will exist a bridged connection between user 1 and user 2, but not for user 3 and user 4. Therefore, there are 7 weakly connected components in the original connected neighborhood (Figure S3 (b)), but when social bridges are considered there will be 6 weakly connected components afterwards (Figure S3 (d)).

For ease of computation, ego users with more than 30,000 followers (i.e., 88 out of 234,834 ego users, less than 0.038% of the overall sample) are omitted in the analysis as the computation of such users would be extraordinarily time consuming. For example, for an ego user with $n = 100,000$ followers, it will involve $n(n-1)/2 = 100,000 \times (100,000-1)/2 = 4,999,950,000$ pairs of Jaccard Similarity computation to determine the existence of bridged connections between the followers. In other words, the computation time it takes will increase in $O(n^2)$. Although social bridges help to capture the implicit structural diversity of individuals, the computational complexity would be a major issue for this approach.

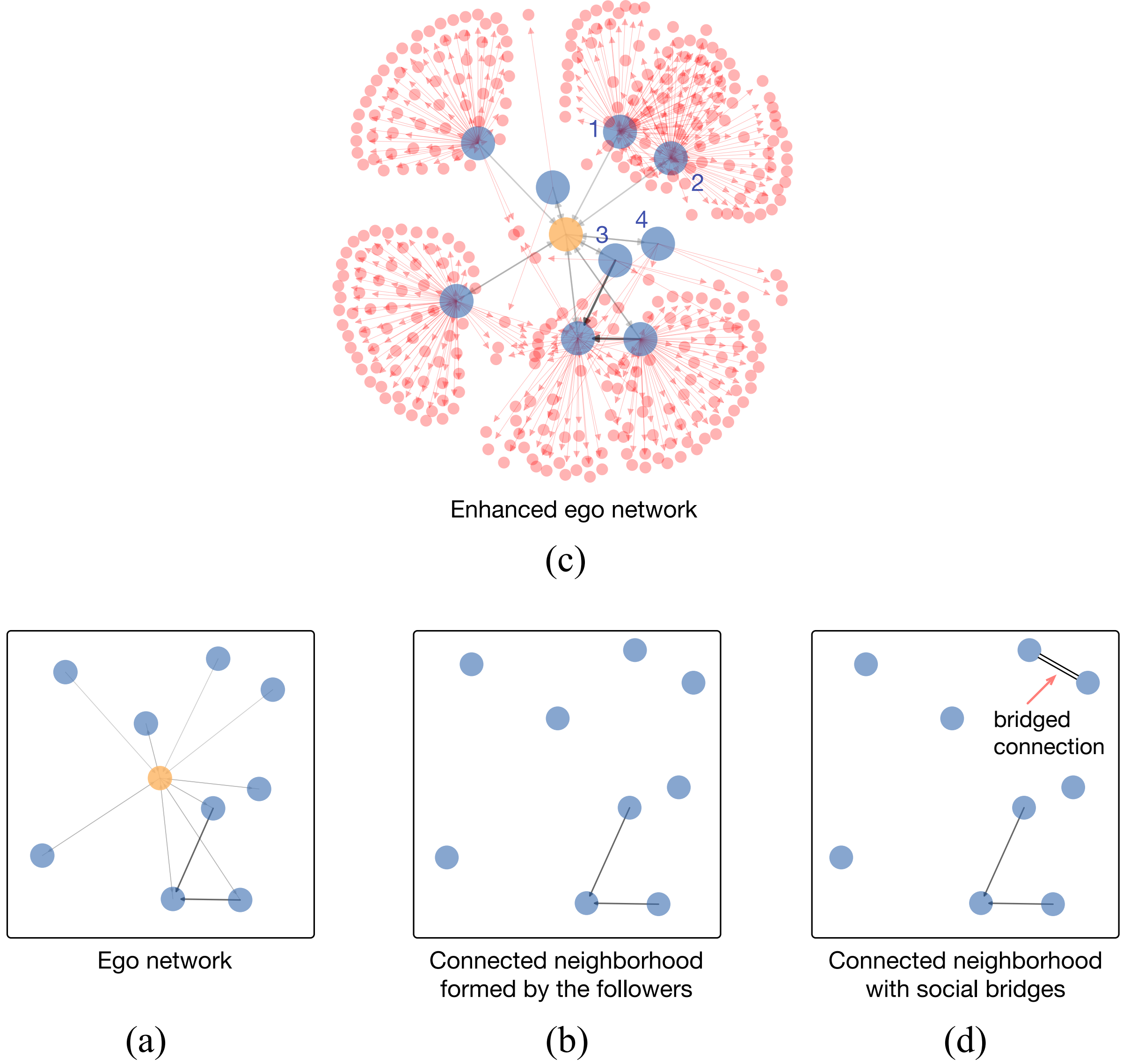

Figure S3. Illustration of the function of social bridges in an example ego network. **(a)** An ego network with the ego user colored in orange and his/her followers colored in blue. **(b)** Connected neighborhood extracted from (**a**) (with the ego user and ties affiliated with the ego user removed). **(c)** Illustration of how social bridges play a role. For the ego user, the followees of his/her followers (geodesic distance = 2 to the ego user) are colored in red. In this example network, user 1 and user 2 have 50 followees in common (including the ego user), and these common followees could act as social bridges between user 1 and user 2. The Jaccard similarity of user 1 and user 2's followees is $JaccardSim(user1, user2) = 0.48$. However, user 3 and user 4 have only 2 followees in common (including the ego user), and the Jaccard similarity of their followees is $JaccardSim(user3, user4) = 0.12$. **(d)** A "bridged connection" (denoted by the double solid line) exists between user 1 and user 2 (when threshold of Jaccard similarity is set as 0.2) due to the function of social bridges between them. Originally, there are 9 nodes and 7 weakly connected components in the original connected neighborhood; but when social bridges are considered, number of weakly connected components becomes 6 (user 1 and user 2 form a new component due to the function of social bridges between them).

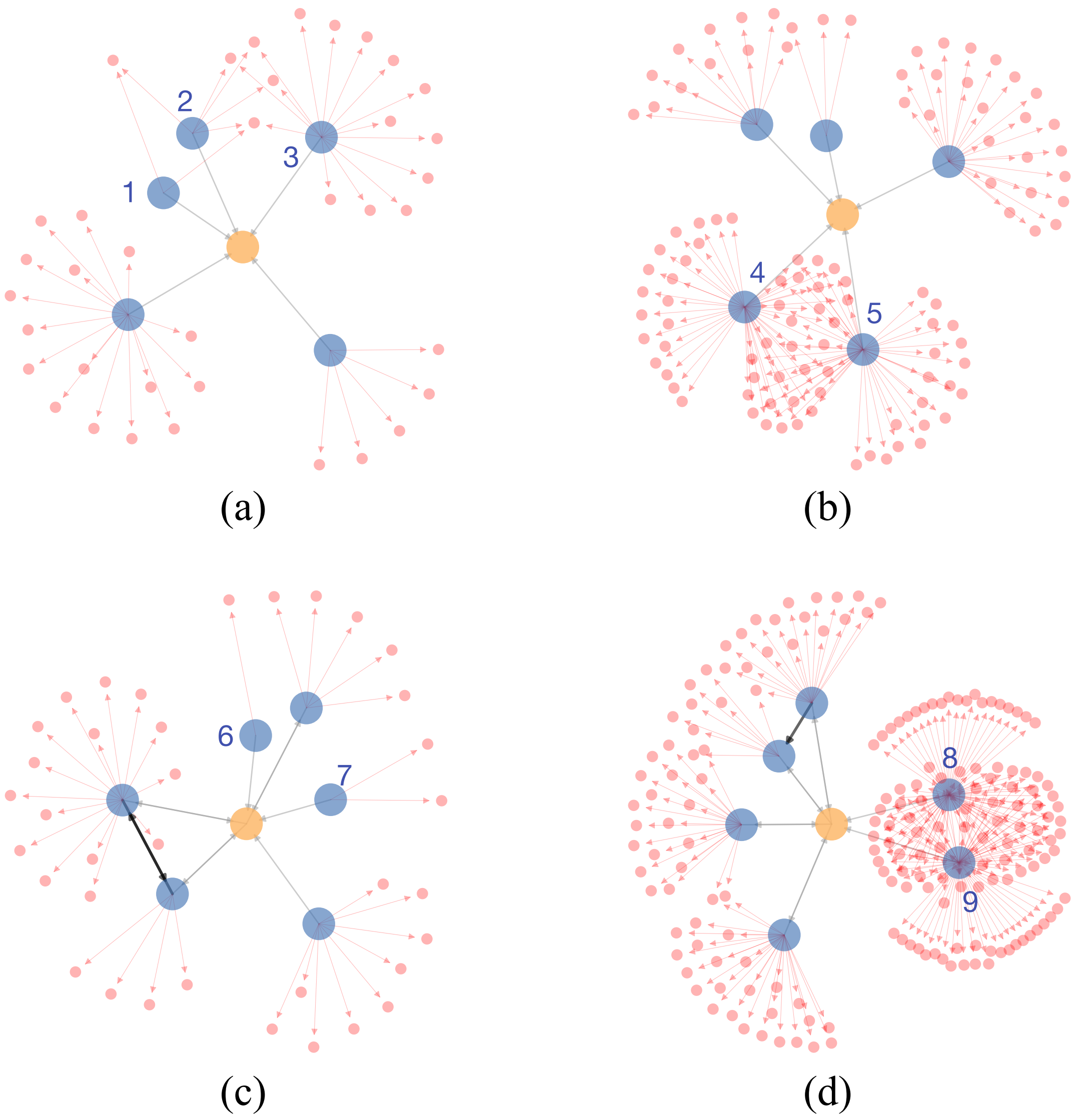

Figure S4. Examples of social bridges in ego networks. (a) An example ego network where no direct social connections exist between any two followers (colored in blue) of the ego user (colored in orange) (i.e., followers are isolated). User 1 and user 2 share a large proportion of followees; user 2 and user 3 also share a large proportion of followees. In this example, diversity measures based exclusively on direct social connections between followers fail to capture the potential structural diversity of the ego user, while social bridges can provide additional power to capture the implicit structural diversity. Specifically, user 1, user 2 and user 3 may belong to one component due to the function of social bridges between them. **(b)** Another example ego network where user 4 and user 5 may belong to one component as they have a large proportion of followees in common. **(c)** Another example network where few social ties exist between followers of the ego user. Although user 6 and user 7 have only one followee in common (which is just the ego user in this case), they may also belong to one component as one common followee still occupies a large proportion of the union of their followees (i.e., Jaccard similarity of their followees is still considerable). **(d)** Another example network where user 8 and user 9 may belong to one component since they have a large proportion of followees in common.

Note 5 - Robustness: controlling possible confounders

Although we have found that for individuals with the same number of followers, those whose followers come from more diverse social backgrounds are likely to have higher social reputations, this finding may be biased by other factors, such as gender and online activity level. To more reliably estimate the statistical effect of structural diversity and eliminate the possible confounding effects induced by other observable characteristics in the prediction of online social reputations, we further collect several types of activity-related data (see Table S3 for the basic descriptions of activity-related data) and conduct matching experiments (see Table S6 for detailed covariates accounted for in matching experiments). We adopt propensity score matching (PSM), a classic approach in matching experiments, in the current study.

We employ a series of matching experiments progressively in terms of whether the quantified diversity measure is larger than or equal to a given threshold. We use the diversity measure via weak connectivity and social bridges to depict the structural diversity of users in the network. For space constraint and simplicity, we only present results when the threshold is set to be in the range [2, 10]. In this scenario, a user is said to be treated (i.e., treatment group) when his/her diversity measure is larger than or equal to m ($m \in [2, 10]$), otherwise untreated (i.e., control group). We do exact matching on indegree and gender and propensity score matching on other covariates. For every treated user in matching experiments, we try to find another untreated user whose indegree and gender are same as the treated user and other activity-related metrics are similar with the treated user using the nearest neighbor search.

After matching, we obtain the relative social reputation index of each matched pair by the difference of the social reputation index of them: $RSRI = SRI_{treated} - SRI_{control}$, where *RSRI* is *Relative Social Reputation Index*; *SRI* is *Social Reputation Index*; and $treated$ and $control$ are treated and control units, respectively. As shown in Figure 6(c) in the main text, treated users have statistically higher social reputations than untreated users, or in other words, users with higher levels of structural diversity (measured by diversity measure via weak connectivity and social bridges) also tend to have higher social reputations. Taken together, matching experiments provide further evidence for the role of structural diversity in accumulating personal online social reputations.

Table S6. Covariates accounted for in propensity score matching. Gender NA means whether a user's gender is undeclared (gender is self-reported on the platform, some users don't disclose their gender at all), while Gender Male means whether a user's gender is male. Variables with highly skewed distributions are log-transformed by $\log_{10}(x+1)$.

Covariates	Matching type
Indegree (log)	exact
Gender (NA)	exact
Gender (Male)	exact
Answer count (log)	propensity score
Question count (log)	propensity score
Article count (log)	propensity score
Column count (log)	propensity score
Pin count (log)	propensity score
Favorite count (log)	propensity score
Followed column count (log)	propensity score
Followed favorite count (log)	propensity score
Followed question count (log)	propensity score
Followed topic count (log)	propensity score
